# Supplementary material for: Microstructural dynamics of motor learning and sleep-dependent consolidation: A diffusion imaging study
Source: iScience. 2023 Nov 10;26(12):108426. doi: 10.1016/j.isci.2023.108426 (PMC10696465; doi:10.1016/j.isci.2023.108426)
Supplement: Document S1. Table S1 [file mmc1.pdf]

iScience, Volume *26*

Supplemental information

Microstructural dynamics of motor learning and sleep-dependent consolidation: A diffusion imaging study

Whitney Stee, Antoine Legouhy, Michele Guerreri, Thomas Villemonteix, Hui Zhang, and Philippe Peigneux

Supplemental Information

Table S1. Participant's demographics, related to Experimental Model and Study Participant Details' section.

	Sleep Deprivation (SD) Group<br>n = 31	Regular Sleep (RS) Group<br>n = 30	Total sample<br>n = 61
Pittsburgh Sleep Quality Index (sleep quality)	mean score = 3.32<br>SD = 1.14<br>min = 1<br>max = 6	mean score = 3.33<br>SD = 1.35<br>min = 0<br>max = 5	mean score = 3.33<br>SD = 1.24<br>min = 0<br>max = 6
Morningness–Eveningness Questionnaire (chronotype)	mean score = 54.71<br>SD = 8.17<br>min = 37<br>max = 73	mean score = 53.42 SD = 7.56<br>min = 32<br>max = 68	mean score = 54.07<br>SD = 7.84<br>min = 32<br>max = 73
Edinburgh Inventory (laterality)	mean score = 5.68<br>SD = 6.16<br>min = -10<br>max = 10	mean score = 4.27<br>SD = 6.64<br>min = -10<br>max = 10	mean score = 4.98<br>SD = 6.39<br>min = −10<br>max = 10
Gender	♀ = 16<br>♂ = 15	♀ = 15<br>♂ = 15	♀ = 31<br>♂ = 30
Age	mean score = 21.07<br>SD = 2.35<br>min = 19<br>max = 29	mean score = 21.57<br>SD = 2.16<br>min = 18<br>max = 29	mean age = 21.31<br>SD = 2.26<br>min = 18<br>max = 29

Legend: Detailed information concerning sleep quality, chronotype, laterality, gender, and age with mean (mean score), standard deviation (SD), minimum (min) and maximum (max) for every group and in the total sample.
